# Supplementary material for: LT-FGRS: a unifying R-package for the estimation of family-based genetic liabilities at population-scale
Source: bioRxiv. 2026 Jun 19:2026.06.15.731517. Preprint. [Version 1] doi: 10.64898/2026.06.15.731517 (PMC13308130; doi:10.64898/2026.06.15.731517)
Supplement: Supplement 1 [file media-1.pdf]

Method	Relatives	Model for right-censoring	Familywise censoring	Original software	LT-FGRS equivalent
**LT-FH / LT-PA**	Only 1st degree	None	-	LT-FH	*get_family_graph(<br>degree = 1)<br>prepare_thresholds(<br>personal_thr = F)<br>estimate_liability(<br>use.mixture = F)*
**PA**	Any	None	-	N/A	*prepare_thresholds(<br>personal_thr = F)<br>estimate_liability(<br>use.mixture = F)*
**$\textbf{PA-FGRS}_{\textbf{mixt}}$**	Any	mixture	-	PA-FGRS	*prepare_thresholds(<br>personal_thr = F)<br>estimate_liability(<br>use.mixture = T)*
**$\textbf{PA-FGRS}_{\textbf{adt}}$**	Any	ADT	-	PA-FGRS	*prepare_thresholds(<br>personal_thr = T,<br>lower_equal_upper = T)<br>estimate_liability(<br>use.mixture = F)*
**LT-FH++ 1.0**	Only 1st degree	ADT	-	LTFHPlus	*get_family_graph(<br>degree = 1)<br>prepare_thresholds(<br>personal_thr = T,<br>lower_equal_upper = T)<br>estimate_liability(<br>use.mixture = F)*
**$\textbf{FGRS}_{\textbf{Kendler}}$**	Any	morbid risk	-	-	kendler_simplified()
**$\textbf{LT-FGRS}_{\textbf{ADT,FW}}$**	Any	ADT	Yes	-	*familywise_censoring()<br>prepare_thresholds(<br>personal_thr = T,<br>lower_equal_upper = T)<br>estimate_liability(<br>use.mixture=F)*
**$\textbf{LT-FGRS}_{\textbf{mixt,FW}}$**	Any	mixture	Yes	-	*familywise_censoring()<br>prepare_thresholds(<br>personal_thr = F)<br>estimate_liability(<br>use.mixture=T)*

Supplementary Table S1. Overview of family-based genetic liability estimation methods and their implementation in LT-FGRS.

Each row corresponds to a method or model variant. **Relatives** indicates whether the method uses only first-degree relatives or any available relative. **Model for right-censoring** describes how partially observed relatives are handled :'None' ignores censoring, 'ADT' applies an age-dependent liability threshold, 'mixture' applies a mixture-based adjustment , and 'morbid risk' uses a weighting based on population morbid risk estimates. **Familywise censoring** indicates whether the method supports restriction of family phenotype records to a specified date, relevant for prospective risk prediction applications. **Original software** lists the previously available implementation, where applicable. **LT-FGRS equivalent** shows the function calls and parameter settings required to reproduce each method within the LT-FGRS package.

Sample	N probands	Average family size	Memory avail.	CPU hours
**iPSYCH (DK)**	141,265	23	20 GB	3
**Norwegian Population Register**	8,966,026	34	217 GB	19

Supplementary Table S2. Computational resource usage when applying LT-FGRS to the iPSYCH and Norwegian Population Register samples.

N probands indicates the number of index individuals for whom LT-FGRS was estimated. **Average family size** reflects the mean number of relatives per proband. Memory and CPU hours reflect observed usage for the runs reported here.

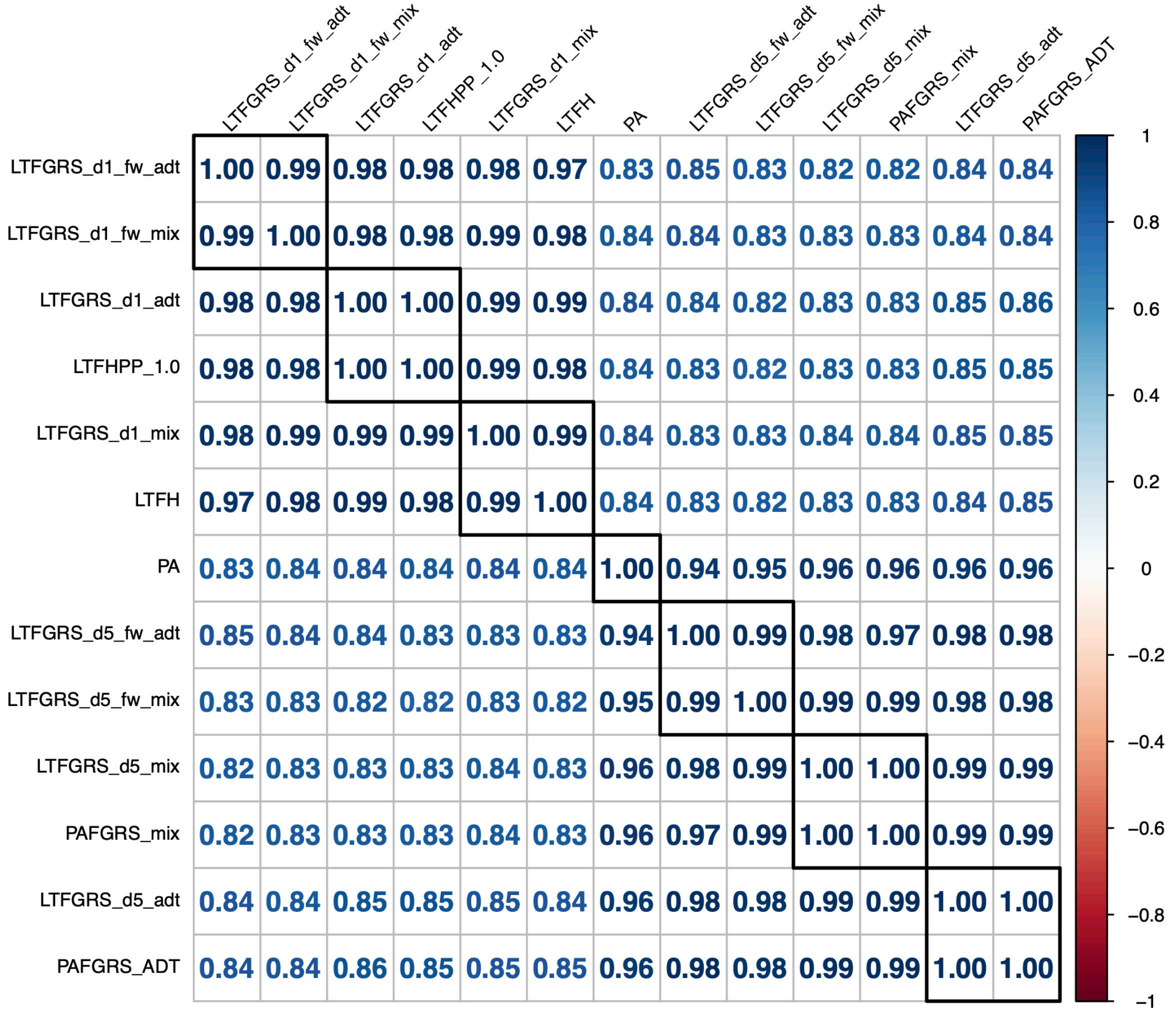

Supplementary Figure S1 | Pairwise Pearson correlations between family genetic risk scores from different methods for major depressive disorder in the iPSYCH sample (N = 141,265).

LT-FGRS are computed using 1st degree relatives only (**_d1**) or up to fifth degree relatives (**_d5**), LT-FGRS is computed without and with familywise censoring – where family members are censored at the date of diagnosis or end of follow-up of the proband (**_fw**). LT-FGRS is computed using two different right-censoring adjustments: the age-dependent-threshold (**_adt**) or the mixture (**_mix**) model. **LTFHPP_1.0** denotes the method from Pedersen et al., **LT-FH** the method from Hujoel *et al.*, **PA** the method from So *et al*., and **PA-FGRS** the method from Krebs *et al.* using the age-dependent-threshold (**_ADT**) or the mixture (**_mix**) model. Black outlines demarcate clusters of methodologically related estimators (using *hclust(k = 7)*).
